# Soil Water Measurement Using Actively Heated Fiber Optics at Field Scale

**DOI:** 10.3390/s18041116

**Published:** 2018-04-06

**Authors:** Duminda N. Vidana Gamage, Asim Biswas, Ian B. Strachan, Viacheslav I. Adamchuk

**Affiliations:** 1 Department of Natural Resource Sciences, McGill University, 21111 Lakeshore Road, Ste-Anne-de-Bellevue, QC H9X 3V9, Canada; duminda.vidanagamage@mail.mcgill.ca (D.N.V.G.); ian.strachan@mcgill.ca (I.B.S.); 2 School of Environmental Sciences, University of Guelph, 50 Stone Road East, Guelph, ON N1G 2W1, Canada; 3 Department of Bioresource Engineering, McGill University, 21111 Lakeshore Road, Ste Anne-de-Bellevue, QC H9X 3V9, Canada; viacheslav.adamchuk@mcgill.ca

**Keywords:** soil water, active heat pulse method, spatial and temporal variation

## Abstract

Several studies have demonstrated the potential of actively heated fiber optics (AHFO) to measure soil water content (SWC) at high spatial and temporal resolutions. This study tested the feasibility of the AHFO technique to measure soil water in the surface soil of a crop grown field over a growing season using an in-situ calibration approach. Heat pulses of five minutes duration were applied at a rate of 7.28 W m^−1^ along eighteen fiber optic cable transects installed at three depths (0.05, 0.10 and 0.20 m) at six-hour intervals. Cumulative temperature increase *(T_cum_)* during heat pulses was calculated at locations along the cable. While predicting commercial sensor measurements, the AHFO showed root mean square errors (RMSE) of 2.8, 3.7 and 3.7% for 0.05, 0.10 and 0.20 m depths, respectively. Further, the coefficients of determination (R^2^) for depth specific relationships were 0.87 (0.05 m depth), 0.46 (0.10 m depth), 0.86 (0.20 m depth) and 0.66 (all depths combined). This study showed a great potential of the AHFO technique to measure soil water at high spatial resolutions (<1 m) and to monitor soil water dynamics of surface soil in a crop grown field over a cropping season with a reasonable compromise between accuracy and practicality.

## 1. Introduction

Soil water is an essential component of many hydrological, climatological and environmental processes. Soil water content (SWC) determines the partitioning of rainfall between infiltration and surface runoff [1,2] and governs the energy fluxes between the land surface and atmosphere through the impact of evapotranspiration and thus affects climatological processes [3]. SWC influences plant growth and ecosystem function and structure [4]; in natural systems, drought can cause large scale and persistent shifts in species distributions. Therefore, characterizing and quantifying the magnitudes and patterns of SWC is essential for a wide range of studies.

SWC varies greatly in space and time due to local (soil properties, topography and vegetation) and non-local (climate) factors influencing it at different intensities at various spatial and temporal scales [5,6,7,8,9,10]. However, the advancement of soil water measurement has limited mostly to point scale using the point–based sensors (e.g., time domain reflectometry [TDR], frequency domain reflectometry [FDR] and capacitance probes) and at large scales using the remote sensing [11]. The scale of soil water measurement is often different from the scale of modeling and this has resulted in a mismatch between the observations and simulations [12,13]. Moreover, modelling of hydrological dynamics has improved significantly, however, measurement capability has not kept pace particularly at intermediate spatial scales such as a field to watersheds [11]. An intermediate spatial scale gap remains where we lack high resolution (both spatial and temporal) SWC data to quantify the patterns and magnitudes of hydrological dynamics and to better assess the performance of various hydrological models [14].

Several methods have emerged to measure SWC at these intermediate scales including Cosmic ray probes [15,16], electromagnetic induction sensors (EMI) [17,18], GPS reflectometry [19] and distributed temperature sensing (DTS) [20,21,22,23]. Among these techniques, DTS is attractive because of its potential to measure soil water at sub-meter intervals along a fiber optic cable up to 10,000 m lengths. This technique can potentially bridge the soil water measurement gap from the point, to field to satellite footprint scales.

There are two categories of DTS method for soil water measurement, namely actively heated fiber optics (AHFO) and passive DTS. AHFO applies an electrically generated heat pulse to the fiber optic cable and the resulting temperature change (thermal response) during or after the heat pulse is related to the water content of the soil using either empirically or physically based equations [21,22]. Passive DTS, on the other hand, uses soil thermal responses to the net solar radiation to estimate soil water [24]. Though the AHFO technique is relatively accurate, only a few studies are available which have evaluated its feasibility to measure SWC at the laboratory scale [20,21,22] and field scale [23,25]. Sayde, Benitez Buelga, Rodriguez-Sinobas, El Khoury, English, van de Giesen and Selker [23] introduced a new empirical calibration function that fits the observed cumulative temperature increase *(T_cum_)* versus SWC in a sand column and they showed the feasibility to measure SWC with a measurement error of lower than 5%. Gil-Rodríguez, Rodríguez-Sinobas, Benítez-Buelga and Sánchez-Calvo [20] used a similar approach and successfully showed the two-dimensional wetting pattern changes in a sandy loam soil column. However, an extension of the AHFO technique to field scale continues to face certain challenges such as field calibration and the power required to heat up longer cables [26]. A field study using drip irrigation by Sayde, Benitez Buelga, Rodriguez-Sinobas, El Khoury, English, van de Giesen and Selker [23] found accurate SWC measurements for the surface layers (30 and 60 cm) when using a calibration (i.e., *T_cum_* vs. SWC) obtained from the same depth range. However, poorer results were obtained from 90 cm depth where the soil texture differed from that in the calibration layer. This identified a limitation of using repacked soil columns for calibration which is not a representative of all soil layers. Moreover, the use of repacked soil columns in the laboratory is tedious and time consuming; to cover a wider moisture range, the column needs to be saturated, drained and dried over several months. Striegl et al. [25] monitored the spatial and temporal dynamics of soil water along a 130 m transect buried in a restoring wetland site. They developed a calibration function relating in-situ measured SWC to the average temperature rise observed from 380 to 580 s after the onset of heating, rather than the *T_cum_* and the fitted function had a RMSE of 1.6% for soil moisture <31% but a RMSE of 5% for wetter conditions.

Despite the limited number of field studies, no studies have tested the feasibility of the AHFO technique using fiber optic cables buried in crop grown soils throughout a cropping season. Two fields studies have shown the feasibility of AHFO technique to measure SWC at deeper soil layers (20–90 cm) [23,25], while its potential to measure SWC in surface soils (<20 cm) remains poorly understood; Perhaps the fiber optic cable buried close to the soil surface can be affected by the atmospheric temperature variations [26]. Understanding the finer scale (<1 m) spatial structure of soil water in crop grown surface soils is important for precision irrigation scheduling [27]. Moreover, a substantial number of point scale SWC measurements in surface soil are required to validate soil moisture products at foot print scale.

The objective of this study was to test the feasibility of the AHFO technique to measure soil water in the surface soil of a crop grown field over a growing season using an in-situ calibration approach. Using an in-situ calibration approach can improve the efficacy of the measurement by integrating a modest network of the point–based sensors at the field scale and expected to minimize errors which can result due to use of repacked soil columns in the laboratory. Overall, results of this study will indicate, if the AHFO technique is a feasible tool for monitoring spatial and temporal soil water dynamics throughout a cropping season at the field scale. 

## 2. Materials and Methods

### 2.1. Site Description 

**Figure 1:**
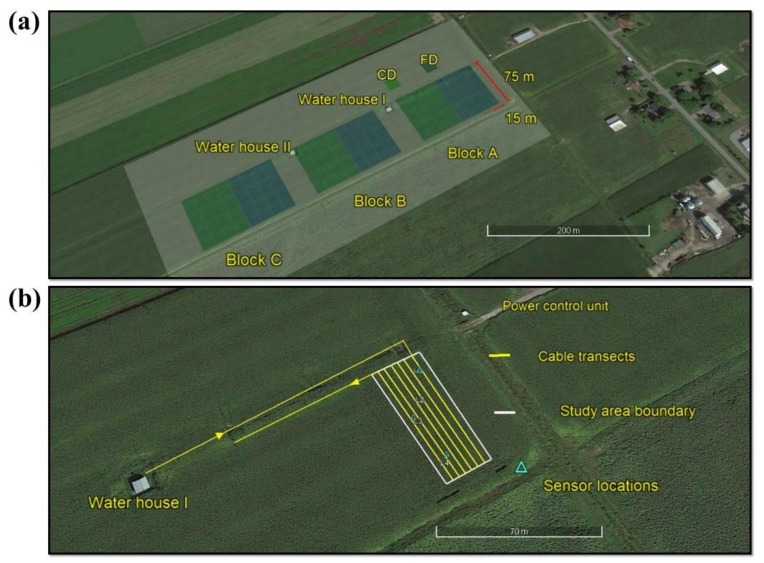
(**a**) Shows the study area which consists of three main blocks A, B and C. Blue and green rectangles within the main block are sub plots (15 × 75 m) of free drainage (FD) and controlled drainage (CD), respectively. Two water houses facilitate the measurement of drainage volume from each sub plot; (**b**) shows the fiber optic cable configuration in the field. Cable connected to the Distributed Temperature Sensing (DTS) instrument starts from the water house I and runs through two subplots at three depths (0.05, 0.10 and 0.20 m), blue rectangles show the locations of 5TE soil moisture sensors used for calibration and validation.

The study site was a 4.2 ha experimental corn field located near Saint Emmanuel, Coteau du Lac, Quebec, Canada (Figure 1a) approximately 60 km west of Montréal. The soil is classified as a Soulanges very fine sandy loam [28], has a mean depth of 0.50–0.90 m and overlies clay deposits from the Champlain Sea. The field has a flat topography, with an average slope of less than 0.5% [29]. The study site consisted of three blocks (A, B and C) and each block comprised eight sub plots (15 m × 75 m). In the center of each sub plot, a tile drain had been installed at 1.0 m depth. These drains discharge into two buildings located on the northern side of the field (Figure 1a). Heating and ventilation help to keep a thermally stable environment inside each building which facilitates year-round measurement of drainage volume. 

### 2.2. Distributed Temperature Sensing System

The DTS system consisted of a DTS instrument and a fiber optic cable connected to it. The DTS instrument used in this study was a Linear Pro series DTS (N4386B, AP Sensing, Böblingen, Germany) and it had two channels with a maximum measurement range of 4 km. The fiber optic cable (BRUsteel, Brugg Cable AG, Brugg, Switzerland) consisted of a stainless steel loose tube containing four multimode 50 μm cores and 125 μm cladding fibers; the steel tube was armored with stainless steel strands and was further enclosed in a protective nylon jacket. The external cable diameter was 3.8 mm. The spatial resolution (integrated length over which a single value of the temperature is recorded by the DTS) and temporal resolution (measurement time frequency) were 0.5 m and 30 s, respectively. 

A laser pulse, generated by the laser source in the DTS instrument travels along the fiber optic cable, a portion of the photons are backscattered and collected by a photon detector in the DTS instrument. Usually, photons are backscattered at the wavelength similar to the wavelength of incident laser pulse which is known as elastic backscattering (Rayleigh scattering). In addition to the elastic backscattering, Raman scattering leads to an inelastic backscattering which results in a return signal of a different wavelength than the incident light. Backscattered photons associated with Rayleigh scattering and Raman scattering are, respectively known as Stokes and Anti-stokes, while the ratio of Stokes to Anti-Stokes is dependent on the temperature. The DTS estimates the temperature using the ratio of intensities of Stokes and Anti-Stokes components and the elapsed time between the laser pulse. Detailed information on the principle of temperature measurement using DTS is well documented by Kurashima et al. [30], while its application for environmental temperature monitoring can be found in Selker et al. [31,32]. 

### 2.3. Fiber Optic Cable Installation 

**Figure 2:**
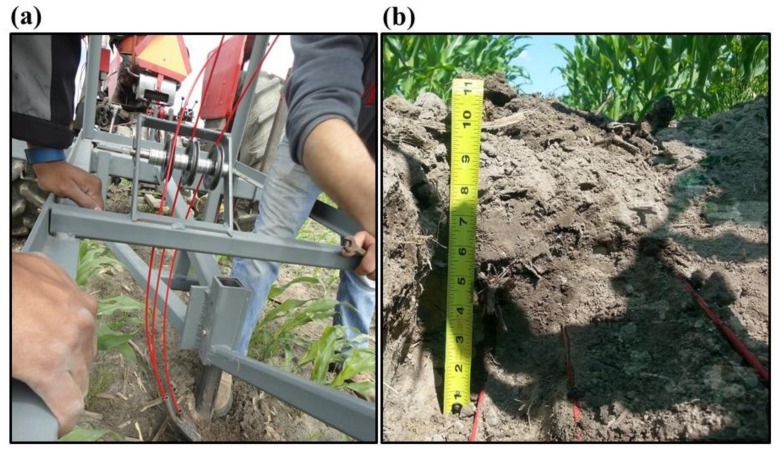
(**a**) Shows the fiber optic cable installation between corn plant rows using the custom plow attached to the back of the tractor; (**b**) shows a section of fiber optic cable installed at 0.05, 0.10 and 0.20 m depths between two corn plant rows. Three cable sections are installed approximately at 45° angle to facilitate fast healing of the cut.

A custom designed plow was used to install the fiber optic cable into 18 transects at three depths 0.05, 0.10 and 0.20 m (6 × 3) in two sub plots of Block A (Figure 1b) on 10 June 2016. Three separated fiber optic cable spools mounted on top the plow were simultaneously fed through three steel tubes attached at the back of the plow blade (Figure 2) in six parallel passes of 73.6 ± 0.3 m long (including the turns). The distance between two successive cable transects was 3.75 m. A duplex single ended calibration set up [33] was achieved by connecting the near cable end (to the DTS instrument) from 0.05 m depth to channel 1 of the DTS instrument, splicing the far cable ends from depths 0.05 and 0.10 m together and splicing the near cable ends from depth 0.10 and 0.20 m together. All the near cable end splices were housed in Splice Box I located inside the water house while all the far end splices were housed in Splice Box II located outside the field. In the duplex single ended configuration, two collocated fibers which were also spliced at the end (Splice box II). This duplex configuration allowed the laser pulse to start from the DTS instrument and travelled through one optical fiber to the Splice box II and from the Splice box II, the laser pulse traveled towards the DTS instrument through the second optical fiber. This allowed two temperature observations at every sampling locations along the fiber optic cable. To calibrate the DTS recorded temperature, three sections of unburied fiber optic cable (20–15 m) were coiled in two cold and one warm, baths. Temperatures of the calibration baths were measured by platinum resistance thermometers (PT100, AP sensing, Germany). Water circulation using aquarium pumps ensured the uniformity of the temperature in each calibration bath. We compared the temperature calibration statistics obtained from DTS inbuilt and the procedure described by Hausner, Suarez, Glander, van de Giesen, Selker and Tyler [33] and presented in Table 1. Calibration statistics such as root mean square error (RMSE) and duplexing error (DE) were estimated for both calibration routines using Equations (1) and (2).

(1) $$\mathrm{RMSE}=\;\frac{1}{n}\sqrt{\overset{n}{\underset{i=1}{\sum }}{\left(T_{i}-T_{\mathrm{oi}}\right)}^{2}}$$

where T_oi_ is the observed independent temperature and T_i_ is the DTS predicted temperature at the i^th^ location of the cable. Number of temperature observations along the calibrated cable section is n.

(2) $$\mathrm{DE}=\;\frac{1}{n}\left|\overset{n}{\underset{i}{\sum }}T_{i1}-T_{i2}\right|$$

Ti_1_ and Ti_2_ are the two temperature observations at the i^th^ cable (same point) and n is total number of temperature observations along the calibrated cable section. The spatial resolution of 0.5 m, 300 s integration time and 30 temperature observation points in the reference cable section were used to calculate RMSE and DE. Mean values of 288 RMSE and DE values calculated at different time periods (August, September and October) and their corresponding standard deviations (SD) are presented in Table 1. User defined calibration included a correction for step loss whereas DTS inbuilt calibration had no correction for step loss prior to calibration. 

### 2.4. Fiber Optic Cable Heating 

**Figure 3:**
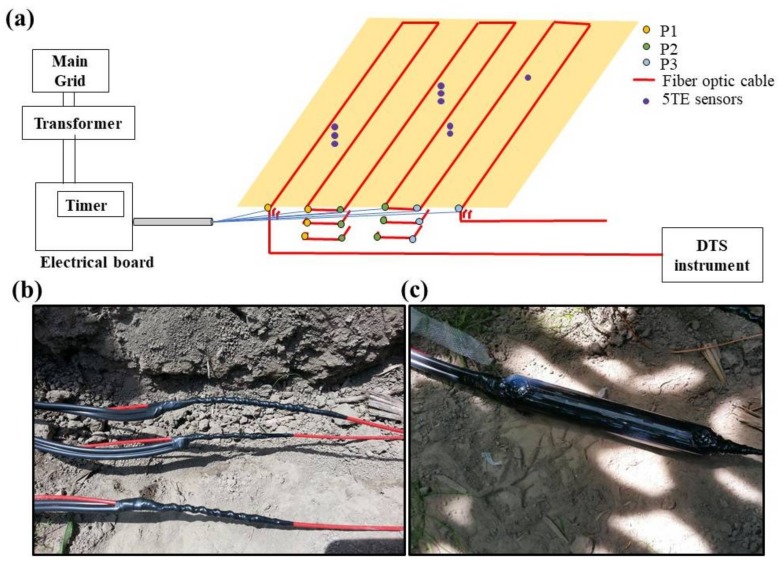
(**a**) Schematic of the power supply from the electrical grid, transformer and main electrical board including the digital timer which controlled the heat pulses (on right), electrical cables pairs connected to each heating cable section (on left); (**b**) shows the electrical connections made to the metal sheath of the fiber optic cable (wrapped with electrical insulating tape); (**c**) an electrical connection enclosed in a plastic container filled with insulating resin.

A domestic electrical grid supplied single-phase electricity to a step-up transformer (15J4Q5D1, Electric Power, Inc., Mississauga, Ontario, Canada) which converted the 120 V to 240 V. After the transformer, a main electrical box including a digital timer (ET1105C, Intermatic, Ontario, CA, Canada) was established. Three pairs of electrical cables (P1, P2 and P3) from the main electrical box were used for heating (Figure 3a). For a given depth, a fiber optic cable section of two consecutive transects was considered as a single heating section. Accordingly, P1, P2 and P3 were connected to heating section 1 (transects 1 and 2), 2 (transects 3 and 4) and 3 (transects 5 and 6), respectively. This allowed the development of fiber optic cable sections (147.3 m long) with similar heating characteristics. At each connecting location, approximately 0.10 m of the protective nylon jacket of the fiber optic cable was removed and electrical cables were connected (Figure 3b). The electrical connections were enclosed in special plastic containers filled with insulating resin (Scotchcast^TM^ 82-A1, Austin, TX, USA) (Figure 3c) to avoid the risk of electrical shock. 240 volts were applied to each heating section to produce heat pulses of 7.28 W m^−1^ that were sent through all the fiber optic cable sections every six hours during a day starting from 12.00 a.m. on the morning of 22 July 2016 to the 6.00 p.m. on the evening of 17 October 2017. A Heating duration of five minutes was used in this study based on the results of previous laboratory experiments which tested both high power–short pulses and low power–long pulses [34]. A digital timer (synchronized with PC time) controlled the start and stop of the heat pulses while both voltage and current intensity were monitored. The DTS instrument (synchronized with PC time) recorded temperature continuously every 30 s along the fiber optic cable (0.25 m sampling interval) during the experimental period. 

### 2.5. Calibration Relationship and Validation

**Figure 4:**
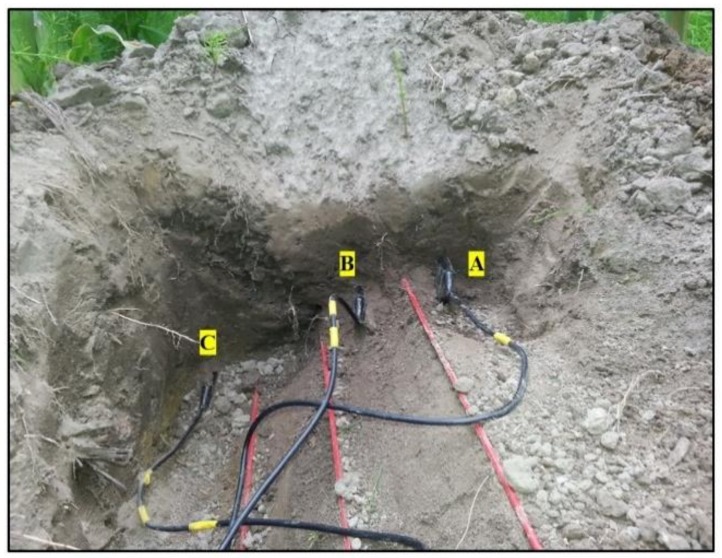
Shows three 5TE soil moisture sensors installed approximately 0.05 m away from the fiber optic cable, A: 0.05m, B: 0.10 m and C: 0.20 m depths in a reference location.

Soil water data for calibration and validation were collected using nine 5TE soil moisture sensors (Decagon Devices, Pullman, WA, USA) calibrated using the gravimetric method installed at 0.05, 0.10 and 0.20 m depths in four reference cable transect locations. At each reference location, the soil was excavated (1 m long × 0.20 m width × 0.25 m depth) and soil moisture sensors were installed approximately 0.05 m away from the fiber optic cables and they had south facing direction at all the reference locations (Figure 4). Before, the excavation, intact soil core samples from the respective depths and locations were collected for soil bulk density and texture determination (Table 2). Ice bags were used to precisely locate 0.5 m long cable sections facing south similar to the installed 5TE soil moisture sensors at each depth within a 1m long reference location (using the thermal signature; cables sections of low temperature). Soil water content at each reference locations was measured every five minutes by 5TE sensors. Only soil water contents measured at corresponding heat pulse times (i.e., 12 a.m., 6 a.m., 12 p.m. and 6 p.m.) were used for calibration and validation. A pair of SWC data corresponding to heat pulse times during a day were randomly selected and this was repeated for all the experimental days to develop a validation data set. The remaining half was treated as the calibration data set. 

### 2.6. Data Analysis 

The integral of the cumulative temperature increase *(T_cum_)* during a heat pulse [21] was calculated at each point of the fiber optic cable using

(3) $$T_{cum}=\overset{t_{0}}{\underset{0}{\int }}∆Tdt$$

and

(4) $$T_{cum\_N}=\frac{T_{cum}}{q}$$

where *T_cum_* is the integral of the cumulative temperature increase (°C s) during the total time of integration *t_0_* (s) at a given point of the cable, ∆T is the DTS recorded temperature change from the pre–pulse temperature (°C). *T_cum_* is a function of soil thermal properties such as the thermal conductivity; higher thermal conductivity (high SWC), will lead to a higher rate at which the heat is conducted away from the cable resulting in a low *T_cum_* at a given point on the cable. In this study, the average temperature calculated over five minutes prior to each heat pulse was used as the pre–pulse temperature. This average was subtracted from the temperature during the pulse to obtain the temperature increase, ΔT. *T_cum_* was then calculated as the sum of the values obtained by multiplying ΔT by the time interval (30 s) between measurements. Accordingly, *T_cum_* was calculated during every heat pulse at each point of the cable transects. *T_cum_* was normalized by power intensity (q) of 7.28 W m^−1^ as *T_cum_N_* using Equation (2). Depth specific calibration relationships were developed using *T_cum_N_* and SWC data obtained only from three reference locations at respective depths (due to a technical problem of soil water sensors at one location, SWC data was used only from three reference locations). SWC data (calibration set; N = 344 for 0.05 m and 0.20 m depths and N = 172 for 0.10 m depth) were obtained from each reference location, while corresponding *T_cum_N_* data were obtained from the 0.5 m cable section which had south facing direction like the 5TE soil moisture sensors (Figure 4). In addition, a single calibration curve was also developed using the *T_cum___N_* and SWC data (N = 860) collected from three depths. Soil water content at each 0.5 m length of the cable transects was subsequently obtained from the T_cum___N_–SWC relationships at respective depths. The AHFO estimated SWC was validated using the 5TE SWC data (validation set). Root mean square error (RMSE) and coefficient of determination (R^2^) were calculated to obtain the averaged predictive accuracy and goodness of fitness, respectively. 

## 3. Results and discussion

### 3.1. Temperature Calibration 

Table 1 clearly indicated that the user defined calibration using reference sections of the fiber optic cable consistently returned the highest accuracies (lowest RMSE) compared to DTS inbuilt calibration routine (Table 1). Unlike the DTS inbuilt calibration, user defined calibration included a step loss correction which resulted in lower DE. It is also important to note that the calibration accuracy was not affected over time; no significant trends and or changes were observed in RMSE values irrespective of the calibration routine. this might be due to the less exposure of the DTS instrument to extreme temperature fluctuations. In this study, we kept the DTS instrument inside a water house which had a thermally stable environment throughout the season in addition to a special out door housing (temperature range from −40 °C to +50 °C) in which the instrument was enclosed.

### 3.2. Calibration and Validation 

**Figure 5:**
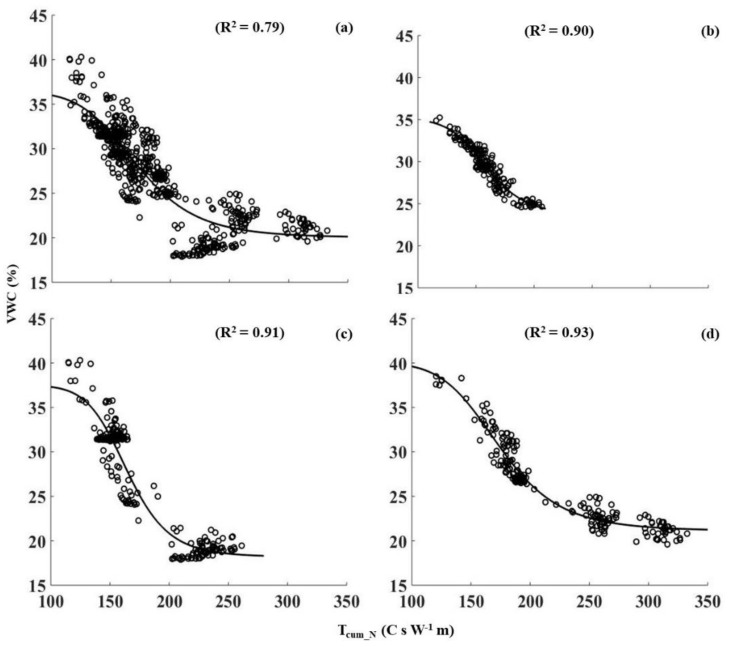
Calibration relationships between soil water content (measured from the 5TE sensors installed near the reference cable locations in the field) and *T_cum___N_* (thermal response measured by the DTS instrument at corresponding cable locations), (**a**) single calibration including all three depths; (**b**) depth 0.05 m; (**c**) 0.1 m and (**d**) 0.20 m depths, respectively.

From this section onwards, SWC is referred to as volumetric water content (VWC). A sigmoidal relationship provided the best fit between and *T_cum___N_* for the depth specific and single calibration relationships (0.05 m: R^2^ = 0.90; 0.10 m: R^2^ = 0.91; 0.20 m: R^2^ = 0.91 and single calibration: R^2^ = 0.79) with RMSE of 0.08, 0.12, 0.18 and 0.02, respectively (Figure 5). The relationship linking the *T_cum___N_* to VWC showed a similar shape for all the curves, particularly for the 0.10 and 0.20 m depths and single calibration exhibited relationships across a wider range of VWC (Figure 5c,d). The sensitivity of *T_cum___N_* increased in dry soil at an increasing rate and the rate decreased after reaching a VWC between 30 and 35% for all the depths (Figure 5). As the water content in the soil surrounding the fiber optic cable increased, heat conduction away from the cable increased because the water’s greater thermal conductivity decreased *_Tcum___N_*. However, as the VWC increased further (>30%), any increase in thermal conductivity was less rapid which results in *T_cum___N_* being less sensitive to actual changes in VWC. Accordingly, the thermal response of the fiber optic cable to the resistive heating yielded similar primary shapes for all the calibration relationships (Figure 5).

**Figure 6:**
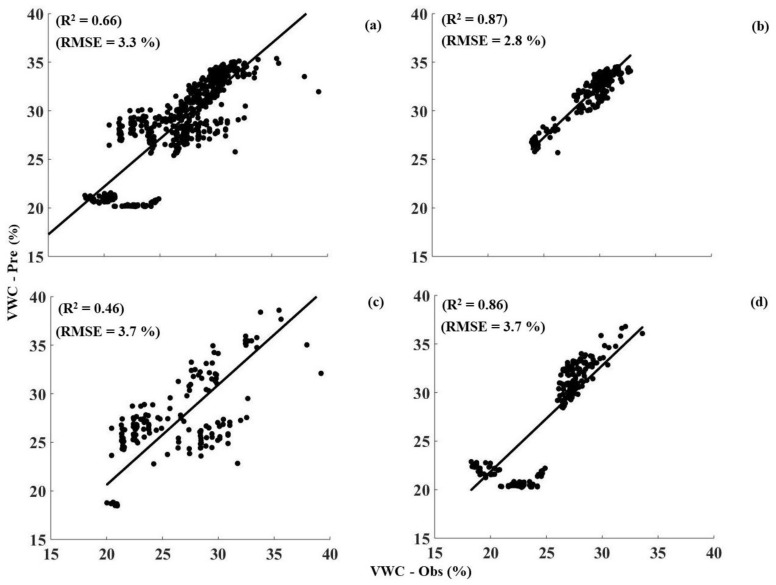
Comparison between observed soil water content by 5TE soil moisture sensors and predicted soil water content by actively heated fiber optics (AHFO) technique (black dots) and black lines are the best fitted lines, (**a**) single calibration including all three depths; (**b**) 0.05 m; (**c**) 0.01 m and (**d**) 0.20 m depths, respectively.

Scattered data points in the single calibration relationship (Figure 5a) indicated the heterogeneity of the *T_cum___N_* under similar soil water contents at different depths (Figure 5a). This was particularly noticeable for the 0.2 m depth; the magnitude of *T_cum___N_* was higher compared to that of at 0.05 and 0.10 m depths under similar water contents (Figure 5b–d). Possible reasons could be the presence of macro pores and/or air gaps at 0.20 m depth; most of the corn roots are distributed within 0.20–0.50 m depths as compared to shallow layers [35] which attributed to a relatively low bulk density (Table 2). Further, soil pore size distribution and bulk densities can be different at reference locations from disturbance during installation. It should be noted that the bulk density values in Table 2 represented the bulk density of locations before excavating. Transient nature of the soil structure healing could also lead to differences in pore size distribution around the cable. This could introduce a transient contact resistance between the soil and the cable [36]. Any decrease or increase in contact resistance might have led to over and under estimation of VWC (Figure 6) Therefore, variations of pore size distribution and bulk densities could account for some of the scatter about the T_cum_N_–VWC relationships (Figure 5). Despite the scattered data points, all the T_cum_N_–VWC relationships were strong, particularly the depth specific T_cum_N_– VWC relationships had the highest coefficient of determinations (R^2^) and used to predict the VWC along the fiber optic cable transects. 

The sensitivity of the calibration curve was dependent on the actual VWC measured by the 5TE soil moisture sensors. Root mean square error of calibrated 5TE soil moisture sensors was 2% which suggested a good measurement accuracy. In comparison to the calibrated 5TE soil moisture sensors, AHFO showed predictive accuracies; RMSE of 3.3, 2.8, 3.7 and, 3.7% for single calibration, 0.05, 0.10 and 0.2 m depths, respectively. Excellent agreements (R^2^ = 0.87 and R^2^ = 0.86) between observed and predicted VWC by the AHFO technique were observed except for 0.10 m depth (R^2^ = 0.46) (Figure 6). It should be noted that a technical problem at one sensor location resulted in a relatively small number of measurement points (N = 172) being used for validation of the 0.10 m depth. Lowest prediction error (RMSE = 2.8%) and highest R^2^ of 0.87 in validation statistics for 0.05 m depth indicated the ability of the AHFO technique to measure VWC in surface soils accurately. It also indicated an advantage of using the *T_cum_* method which was corrected for pre-pulse or back ground temperature (Equation (1).

Since remote sensing measurements are at scales of kilometers, a substantial number of point scale soil water measurements are required to validate soil water measurements due to the spatial variability at foot print scales. The AHFO technique can potentially measure soil water across a lager spatial support (e.g., >1 km) [37]. Results of this study also showed that the AHFO technique could measure soil water along a 1300 m long fiber optic cable which is a relatively larger spatial support. Therefore, AHFO technique has a great potential to support in validating satellite moisture products by reducing the contrast between the spatial support of ground-based observations and satellite-based soil moisture estimations. Moreover, the AHFO technique can be potentially used in a soil water upscaling technique such as temporal stability to find the locations that best represent the aerial mean soil water content [38,39,40]. This can reduce the number of point scale measurements required to monitor soil water content at foot print scales. Bhatti et al. [41] showed that the assessment by the temporal stability concept proved to be useful and results suggest that probe measurements at 0.10 m depth best matched to the satellite observations. 

### 3.3. Monitoring Variability of Soil Water Using AHFO Technique 

**Figure 7:**
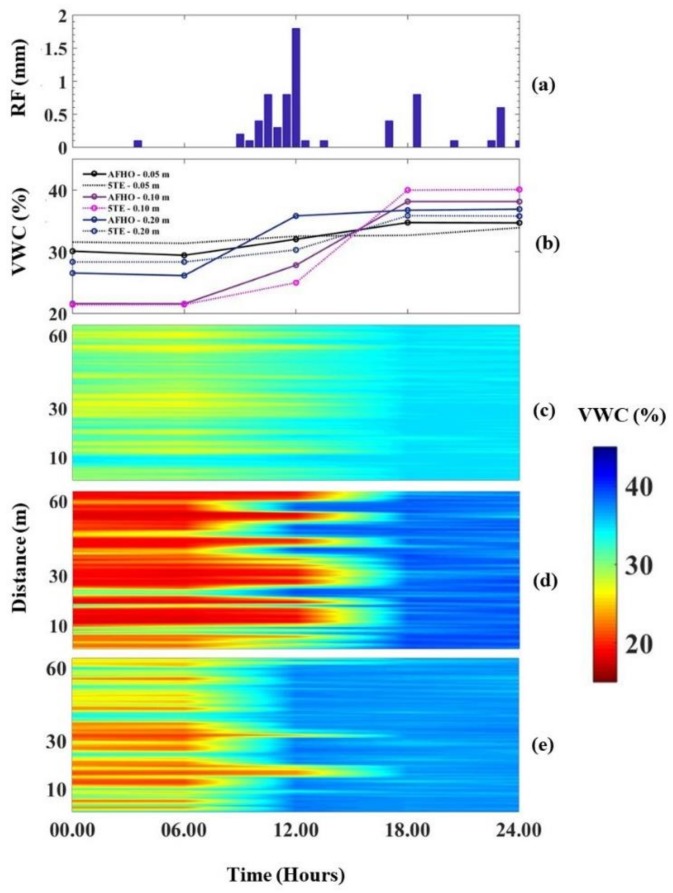
(**a**) Shows the pattern of rainfall during the period from 12 a.m. 13th August 2016 to 12 a.m. 14th August 2016 (24 h period); (**b**) shows the temporal changes in mean soil water content of transect 1 and soil water content measured by the 5TE sensors at three depths during the similar period, (**c**–**e**) shows the changes of soil water content of the transect 1 predicted by AHFO technique at 0.05, 0.10 and 0.20 m depths along the transect 1 (distance in meters), respectively.

**Figure 8:**
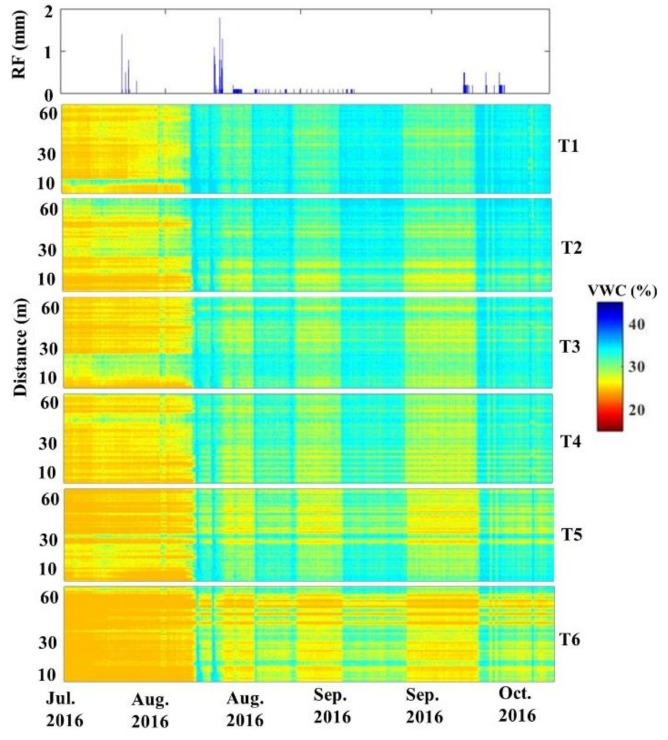
Variation of soil water content at every 0.5 m (location) along the six transects (T1 to T6) during the period from 22nd July 2016 to 17th October 2016 in response to rainfall at 0.05 m depth.

To examine the feasibility of the AHFO technique to detect the soil water changes at the field scale, we explored the ability of the AHFO technique to detect rainwater infiltration into the soil within a selected 24–h period (12.00 a.m. 13 August–12.00 a.m. 14 August 2016; Figure 7). VWC predicted by the AHFO technique showed good agreement with rainfall events. VWC at 0.05 m depth showed that a rapid increase in response to rainfall occurred between 6 a.m. and 12 p.m. (Figure 7c). Corresponding cable locations of the 0.10 m depth clearly showed a time lagged increase in VWC (Figure 7d). The deeper layer (0.20 m) was relatively wetter than the 0.10 m layer during the dry period (from 12 a.m. to 6 a.m. 13th August 2016) and this resulted in no visible time lagged increase in VWC at the deeper layer. Moreover, there was a rainfall event of <0.5 mm during the time from 12 a.m. to 6 a.m. (Figure 7a), however, it was not sufficient to increase the VWC in 0.10 and 0.20 m depths while a little increase in VWC was visible in 0.05 m depth (Figure 7c). These results suggested that the fiber optic cable could respond to small variations in VWC of surface soil. Marked spatial variation in VWC along the transect was found in the shallow (0.05 m) layer compared to the deeper layers. Furthermore, the temporal patterns of VWC measured from the 5TE sensors and the mean VWC across the transect from the AHFO technique are nearly identical, despite their different measurement volumes (Figure 7b). This agreement showed that the AHFO technique could reliably track soil water changes and demonstrated the feasibility of using this technique at the field scale. While quantification of spatial and temporal dynamics of soil water is beyond the scope of this paper, VWC predicted from the AHFO technique displayed a positive correlation with precipitation, as expected (Figure 8). The surface soil was drier during the period from July to mid-August 2016 which could be attributed to a lack of rainfall and high evaporation from the soil during the early growth of the corn. These findings were consistent with those of other studies [42,43]. VWC was comparatively higher and mostly related to the rainfall events that occurred during the period from mid-August to mid-October 2016 (Figure 8). In addition, a soil water gradient from transect 1 to 6 was evident during the same period which demonstrated the variation in the distribution of surface soil water after rainfall events. However, more analysis is necessary to interpret the observed spatial and temporal variations of VWC. 

### 3.4. Challenge of Field Calibration 

In this study, we tested an in-situ calibration methodology relating the DTS observed *T_cum_* and VWC measured by calibrated 5TE soil moisture sensors in a crop grown field. The AHFO predicted VWC in a cropped field using the depth specific calibration relationships. The accuracy of the in-situ calibration could be affected by several factors. Dispersion between observed and the predicted VWC across all three depths can be attributed to the mismatch between support of the measurement by the two methods. Support is the area or volume of soil over which the measurement is integrated by the sensor [44]. Support of 5TE sensor was approximately 0.715 L [45]. There are no data yet available on the volume of soil influenced by the fiber optic cable section (e.g., 0.5 m used in this study). Further, spatial variability of soil thermal properties (effects of soil texture and organic matter) can affect the variability of thermal response under similar VWC. Usowicz et al. [46] found a distinct impact of bulk density on the spatial variability of soil thermal properties in addition to soil water content in corn cultivated fields while Abu-Hamdeh and Reeder [47] showed that the changes in soil organic matter and texture affected the thermal conductivity measured in repacked soils in the laboratory. Presence of macro pores, roots (in cropped soils) and may also affect the soil water transport near the 5TE soil moisture sensors [48] and temperature measurement by the fiber optic cables. Field installation of fiber optic cable at desired depths with a good soil contact and minimal disturbance is also a challenge. Studies have used custom designed plows [23] and commercial vibratory plows [25]. We also used a custom designed plow and started heat pulse measurement after five weeks of cable installation to provide a sufficient time for healing the soil cut. Transient nature of the contact resistance between the cable and the soil could impact the soil water measurements [36]. Future research is needed to determine the impact of thermal contact resistant on soil water measurement in different soils. Though we used calibrated temperature to estimate the soil water in this study, it might not be required as the AHFO technique uses the relative temperature change rather the absolute temperature. This is particularly true for *T_cum_* method, as it calculates the integral of the temperature change during the heating phase. However, it would be worth to compare the accuracy of soil water estimated using both calibrated and non–calibrated temperature. 

## 4. Conclusions

This study investigated the feasibility of the AHFO technique to measure soil water in the surface soil of a crop grown field over a growing season using an in-situ calibration approach. Depth specific strong calibration relationships between SWC and *T_cum_N_* predicted SWC with satisfactory accuracy when compared with calibrated commercial soil water sensors (RMSE = 2.8, 3.7 and 3.7% respectively). Further, strong agreements between AHFO predicted and 5TE sensors measured SWC were found at depths. The AHFO technique could accurately monitor the normal SWC variations of surface soil resulting from rainfall at diurnal to seasonal scales in a cropped field. Use of an in-situ calibration approach improved the efficacy of the measurement by integrating a modest network of point–based sensors at the field scale. Overall, this study showed a great potential of the AHFO technique to measure soil water at high spatial resolutions (<1 m) and to monitor soil water dynamics of surface soil in a crop grown field over a cropping season with a reasonable compromise between accuracy and practicality. 

## Figures and Tables

**Table 1:** Calibration statistics of RMSE and DE estimated at different time periods.

Calibration Routine	DTS Inbuilt	User Defined	DTS Inbuilt	User Defined
Time period	RMSE (°C) Mean ± SD	RMSE (°C) Mean ± SD	DE (°C) Mean ± SD	DE (°C) Mean ± SD
August	0.65 ± 0.07	0.17 ± 0.04	0.21 ± 0.06	0.10 ± 0.04
September	0.59 ± 0.09	0.11 ± 0.08	0.17 ± 0.08	0.07 ± 0.02
October	0.62 ± 0.05	0.15 ± 0.03	0.19 ± 0.04	0.08 ± 0.03

RMSE: root mean square error, DE: duplexing error and SD: standard deviation.

**Table 2:** Measured soil properties in the study area at reference locations at depths.

Soil Property	0.05 m	0.10 m	0.20 m
Clay (%)	9.52 ± 1.51 *	10.70 ± 0.01 *	10.09 ± 0.02 *
Silt (%)	41.52 ± 24 *	40.13 ± 2.68 *	39.53 ± 1.38 *
Sand (%)	48.96 ± 1.57 *	49.18 ± 2.68 *	50.38 ± 1.36 *
Textural class	Loam	Loam	Loam
Bulk density (Mg^−3^)	1.44 ± 0.21 *	1.41 ± 0.15 *	1.37 ± 0.12 *

* Standard deviation of four replicates.
